# Wilkie's Syndrome as a Rare Cause of Duodenal Obstruction: Perspicacity Is in the Radiological Details

**DOI:** 10.7759/cureus.10467

**Published:** 2020-09-15

**Authors:** Ahmad A Al Faqeeh, Muhammad Khalid Syed, Mohammed Ammar, Talal Almas, Saifullah Syed

**Affiliations:** 1 Pediatric Surgery, King Fahad Hospital, Al Baha, SAU; 2 Internal Medicine, Royal College of Surgeons in Ireland, Dublin, IRL

**Keywords:** wilkie's syndrome, gastrointestinal tract, obstruction

## Abstract

Superior mesenteric artery syndrome, or Wilkie's syndrome, is an unexpected cause of upper gastrointestinal tract obstruction. The exact incidence of the condition remains unknown, and limited case reports are present in the literature. The obstruction results in the compression of the third part of the duodenum between the superior mesenteric artery and aorta. It is widely known that a lack of subcutaneous tissue in the area can precipitate the obstruction by significantly reducing the aortomesenteric angle. Wilkie’s syndrome presents a clinically diagnostic challenge as patients initially remain undiagnosed with relapsing episodes of upper abdominal pain and bilious vomiting. In some cases, an acute obstruction may arise. Undertaking an initial contrast study of the upper gastrointestinal tract and a CT scan are required to confirm the diagnosis of the condition. In the present study, we elucidate the case of a 12-year-old girl who presented with upper abdominal pain and bouts of bilious vomiting. Upon extensive diagnostic evaluation, Wilkie’s syndrome was diagnosed. Since the patient failed to respond to conservative treatment, a laparotomy with subsequent duodenojejunostomy was undertaken. The postoperative recovery of the patient was uneventful with no recurrence of symptoms on follow-up.

## Introduction

Superior mesenteric artery syndrome (SMAS), or Wilkie's syndrome, forms a rare but intriguing cause of upper gastrointestinal tract (GIT) obstruction. The incidence in the general population hovers between 0.013% and 0.78% [1]. In 1927, Wilkie described the detailed pathophysiology and management of SMAS [2]. The superior mesenteric artery (SMA), upon branching from the aorta, crosses the third part of the duodenum. The fat and lymphatic tissue protects the duodenum from compression by the SMA. SMAS poses a significant diagnostic challenge, with radiology forming the basis of majority of the diagnoses of SMAS. Additionally, recurrent admission due to the relapsing and remitting course of symptoms makes the diagnosis very difficult [3]. In this paper, we delineate a rare instance of Wilkie’s syndrome in a 12-year-old female patient.

## Case presentation

**Figure 1:**
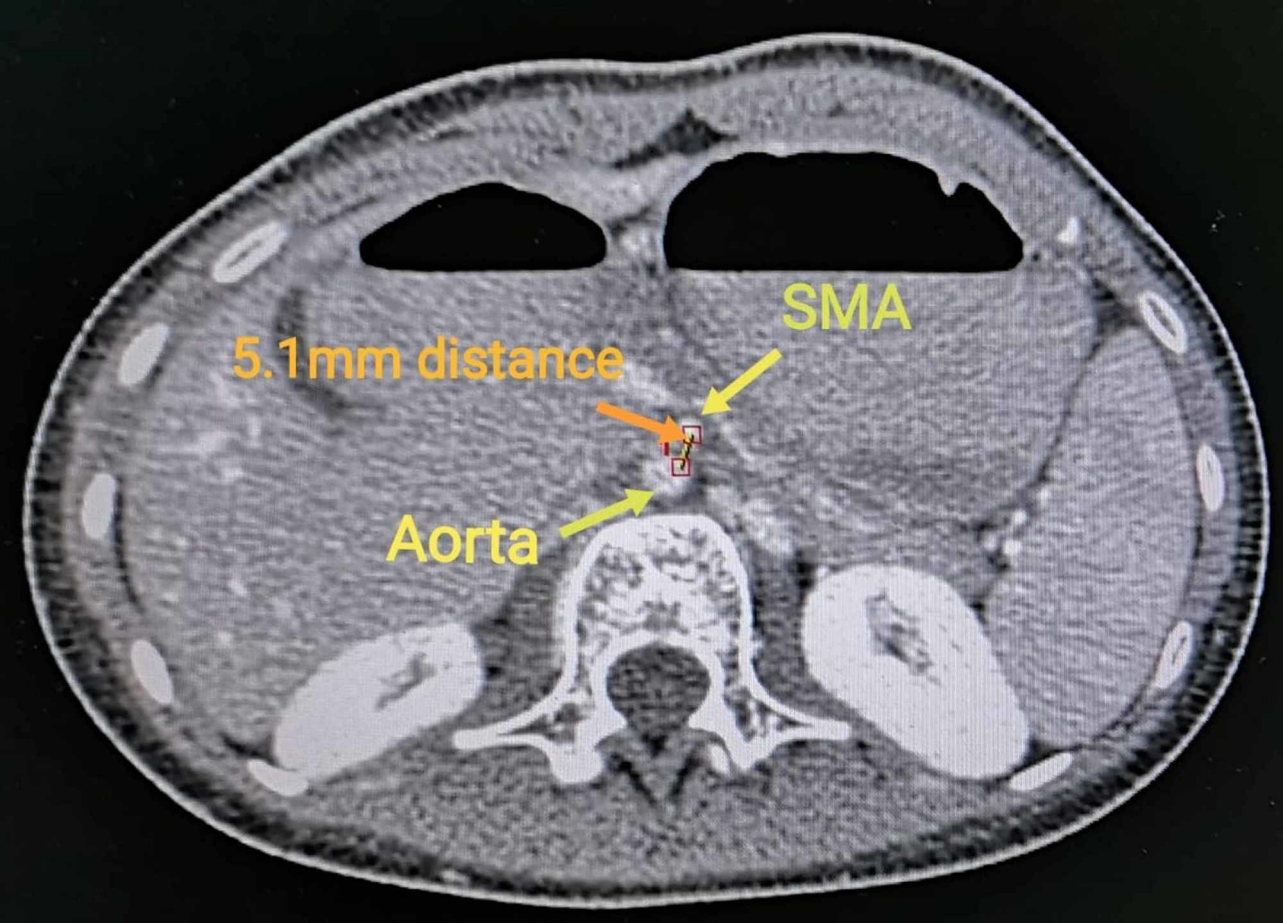
CT scan showing a reduced distance of 5.1 mm (normal 10-20 mm) between SMA and aorta, insinuating a diagnosis of superior mesenteric artery syndrome. SMA: superior mesenteric artery

A 12-year-old female presented to our department with a history of recurrent upper abdominal pain and bilious vomiting. The relapsing and remitting course of the patient’s symptoms resulted in a history of multiple hospital admissions with subsequent attempts at conservative management through nasogastric tube aspiration, intravenous fluids, analgesics, and prophylactic antibiotics. Physical examination findings revealed a thin, underweight child with a congenital web neck and mild upper abdominal distention and diffuse abdominal tenderness. A contrast radiograph demonstrated a dilated stomach with limited gas present in the rest of the small intestine, alluding to an upper GIT obstruction. The proximal portion of the duodenum was also visibly dilated with no contrast reaching the jejunum. A CT scan of the abdomen showed compression of the third part of the duodenum and a reduced aortomesenteric distance (Figure 1). 

**Figure 2:**
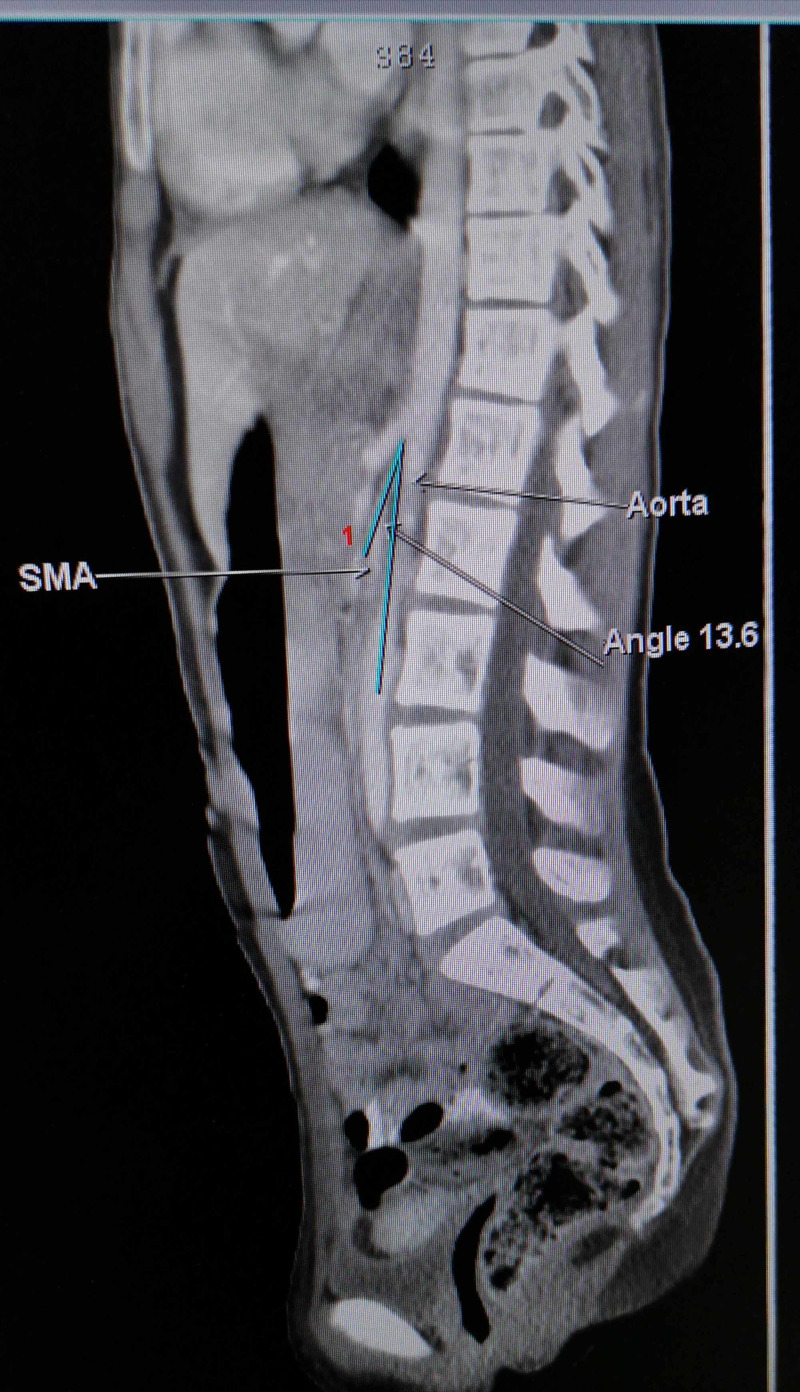
CT scan showing a reduced aortomesenteric angle, measuring 13.6° (normal 25°-60°), reaffirming the diagnosis of Wilkie’s syndrome. SMA: superior mesenteric artery

Additionally, the CT scan divulged a narrow aortomesenteric angle as expected in a patient with Wilkie's syndrome (Figure 2).

Despite being conservatively managed for seven days by nasogastric tube aspiration, intravenous fluids, analgesics, and prophylactic antibiotics, the patient’s clinical picture did not improve. On the eighth day of admission, an exploratory laparotomy with an upper midline incision was performed to better elucidate the etiology underlying the patient's symptoms. A duodenojejunostomy, with displacement of a loop of the jejunum 20 cm distal to the duodenojejunal junction passing the transverse mesocolon, was then performed. Finally, a side-to-side anastomosis was made with the first part of the duodenum. Until 10 days postoperatively, the patient remained on intravenous fluids in order to avoid a recurrence or exacerbation of symptoms. The patient was discharged on the 12th postoperative day. Thereafter, the patient was managed conservatively and recovered promptly. Upon six months of follow-up, the patient remained free of symptoms.

## Discussion

Wilkie’s syndrome is considered an exceedingly rare presentation in the pediatric population [1,4]. Our case depicts a rare instance of the condition in a 12-year-old female. The normal aortomesenteric angle and distance are 25°-60° and 10-20 mm, respectively [4]. If a patient has experienced a significant reduction in weight, the aortomesenteric angle reduces from the normal of 25°-60° to between 6° and 15° and the aortomesenteric distance reduces to 2-8 mm, resulting in compression of the duodenum [3,4]. The diagnostic challenge that SMAS poses clinically can be attributed to a number of factors. These factors include the presence of vague signs and symptoms, such as upper abdominal pain, bilious vomiting, and weight loss, as well as the possibility of a variable age at presentation. Recurrent admission and improvement on conservative treatment further contributes to this diagnostic challenge [4]. Contrast studies of the upper GIT in patients with Wilkie's syndrome show a dilation of the stomach, first and second part of the duodenum, and visible compression of the third part of the duodenum [5,6]. The diagnosis is confirmed by a CT scan with contrast, which demonstrates the compression present in the third part of the duodenum and thus highlights the reduction in the aortomesenteric distance [5]. Radiology plays a pivotal role in the diagnosis of 90% of SMAS cases, manifesting a high sensitivity for diagnosing the condition [7]. Conservative treatment, although successful in some patients, lends to the possibility of recurring symptoms that subsequently mandate surgical intervention. It includes adopting a nil per os (NPO) approach with nasogastric tube insertion as well as positioning the patient laterally. Although not employed in our case, total parenteral nutrition (TPN) is also considered in some cases [6,7].

In the instance of an unsuccessful clinical response to conservative treatment, surgical intervention is considered. Duodenojejunostomy is usually considered as the surgical modality of choice. In this procedure, a loop of the jejunum is anastomosed with the second part of the duodenum. This surgery generally yields good postoperative outcomes, including complete remission of symptoms and relief from obstruction. Other possible procedures that can be performed include a gastrojejunostomy and Roux-en-Y duodenojejunostomy [8,9]. Notably, greater than 75% of patients eventually require surgical intervention [9]. Additionally, laparoscopic duodenojejunostomy is another well-established and less invasive surgical approach that affords a 90% success rate [10]. Due to the severity of the patient's symptoms, this less invasive approach was not employed in our case.

Even with the advent of novel conservative regimens, surgical intervention is most often imperative. A lack of guidelines remains a roadblock for the timely diagnosis and prompt treatment of Wilkie’s syndrome. This is mainly due to the paucity of data and low prevalence rate, especially in the pediatric population. The development of appropriate guidelines and further advancements in minimally invasive surgery will improve the overall quality of life in such patients.

## Conclusions

Wilkie’s syndrome, being a rare cause of upper GIT obstruction in the pediatric age group, ultimately requires surgery in order to yield favorable outcomes including complete remission of symptoms. While conservative management may play a role in relieving symptoms in the early stages of a mild obstruction, there remains an unmet need for the curation of a set of guidelines to promptly diagnose this aberrant anatomical pathology. Since a delayed diagnosis may eventually result in increased mortality rates, the prompt diagnosis of this ailment through meticulous radiological imaging and clinical evaluation remains pivotal. 
